# Characterisation of a novel nucleorhabdovirus infecting alfalfa (*Medicago sativa*)

**DOI:** 10.1186/s12985-019-1147-3

**Published:** 2019-04-29

**Authors:** Yahya Z. A. Gaafar, Katja R. Richert-Pöggeler, Christina Maaß, Heinrich-Josef Vetten, Heiko Ziebell

**Affiliations:** 1 Julius Kühn Institute, Institute for Epidemiology and Pathogen Diagnostics, Messeweg 11-12, 38104 Braunschweig, Germany; 2 Cremlingen, Germany

**Keywords:** electron microscopy, high throughput sequencing, lucerne, rhabdovirus, alfalfa-associated nucleorhabdovirus

## Abstract

**Background:**

Nucleorhabdoviruses possess bacilliform particles which contain a single-stranded negative-sense RNA genome. They replicate and mature in the nucleus of infected cells. Together with viruses of three other genera of the family *Rhabdoviridae*, they are known to infect plants and can be transmitted by arthropod vectors, during vegetative propagation, or by mechanical means. In 2010, an alfalfa (*Medicago sativa*) plant showing virus-like symptoms was collected from Stadl-Paura, Austria and sent to Julius Kühn Institute for analysis.

**Methods:**

Electron microscopy (EM) of leaf extracts from infected plants revealed the presence of rhabdovirus-like particles and was further used for ultrastructural analyses of infected plant tissue. Partially-purified preparations of rhabdovirus nucleocapsids were used for raising an antiserum. To determine the virus genome sequence, high throughput sequencing (HTS) was performed. RT-PCR primers were designed to confirm virus infection and to be used as a diagnostic tool.

**Results:**

EM revealed bacilliform virions resembling those of plant-infecting rhabdoviruses. HTS of ribosomal RNA-depleted total RNA extracts revealed a consensus sequence consisting of 13,875 nucleotides (nt) and containing seven open reading frames (ORFs). Homology and phylogenetic analyses suggest that this virus isolate represents a new species of the genus *Nucleorhabdovirus* (family *Rhabdoviridae*). Since the virus originated from an alfalfa plant in Austria, the name alfalfa-associated nucleorhabdovirus (AaNV) is proposed. Viroplasms (Vp) and budding virions were observed in the nuclei of infected cells by EM, thus confirming its taxonomic assignment based on sequence data.

**Conclusions:**

In this study, we identified and characterised a new nucleorhabdovirus from alfalfa. It shared only 39.8% nucleotide sequence identity with its closest known relative, black currant-associated rhabdovirus 1. The virus contains an additional open reading frame (accessory gene) with unknown function, located between the matrix protein and the glycoprotein genes. Serological and molecular diagnostic assays were designed for future screening of field samples. Further studies are needed to identify other natural hosts and potential vectors.

**Electronic supplementary material:**

The online version of this article (10.1186/s12985-019-1147-3) contains supplementary material, which is available to authorized users.

## Background

Alfalfa or lucerne (*Medicago sativa* L.), a member of the *Fabaceae* family, is used as perennial forage crop which is important as fodder for livestock, as green manure for soil fertility, and can be used as food and medicine for humans [1–4]. It is grown worldwide in temperate zones. Similar to other legumes, alfalfa is susceptible to a range of pests and pathogens [5]. Alfalfa can be infected by a large number of viruses such as alfalfa mosaic virus (AMV) (family: *Bromoviridae*) and two rhabdoviruses (alfalfa dwarf virus (ADV) and lucerne enation virus (LEV)) [6–9].

Members of the *Rhabdoviridae* family (order *Mononegavirales*) infect humans, invertebrates, vertebrates and plants [8, 10–12]. Typically, their virions are bacilliform or bullet-shaped, composed of a helical nucleocapsid coated by a matrix layer and a lipid envelope while some have non-enveloped filamentous virions. The family has eighteen genera including 135 assigned species [13]. Sixteen genera have a monopartite genome while two are bipartite. Their genomes are linear and consist of negative-sense, single-stranded RNA (−ssRNA) (11–16 kb in length) and can comprise up to ten or more genes. They have five canonical genes that may be overprinted, overlapped or interspersed with additional accessory genes [14–16]. Viruses of the genera *Cytorhabdovirus*, *Dichorhavirus*, *Nucleorhabdovirus* and *Varicosavirus* are known to infect plants [17].

The genus *Nucleorhabdovirus* has currently ten assigned species. Nucleorhabdoviruses are known to be transmitted by leafhoppers (*Cicadellidae*), planthoppers (*Delphacidae*) and aphids (*Aphididae*) [17–19]. Additionally, some can also be transmitted during vegetative propagation or by mechanical means. They can replicate in both plants and insect vectors [20]. In plant cells, they replicate in the nucleus which becomes enlarged and develops large granular nuclear inclusions. They have non-segmented genomes, and like other rhabdoviruses they have highly conserved regulatory regions separating their genes, and complementary 3′ leader (l) and 5′ trailer (t) sequences. The 3’l and 5’t complementary sequence has the ability to form a putative panhandle structure suggested to be involved in genome replication [21].

With the advances in molecular techniques and bioinformatic tools, several new members of the *Rhabdoviridae* have been identified recently [22–26]. In this study, we succeeded in sap transmission of a rhabdovirus from *M. sativa* to *Nicotiana benthamiana* and identified it as a hitherto undescribed nucleorhabdovirus for which we propose the tentative name alfalfa-associated nucleorhabdovirus (AaNV).

## Methods

### Sample source and virus isolates used

During a survey in Stadl-Paura (Austria) in May 2010, a sample was collected by Dr. Herbert Huss from an alfalfa plant showing virus-like symptoms (symptoms were not recorded at the time) and sent to Julius Kühn Institute (JKI) for analysis. In initial attempts at virus isolation by sap transmission, the putative virus was transmitted to *N. benthamiana* seedlings as described below for further analysis and virus propagation (JKI ID 24249). For comparative studies, physostegia chlorotic mottle virus (PhCMoV; JKI ID 26372) and eggplant mottled dwarf virus (EMDV; JKI ID 29094) were maintained on *N. benthamiana* under greenhouse conditions by serial mechanical transmission.

### Electron microscopy

For electron microscopy, small pieces (ca. 5 mm in diameter) of symptomatic leaves from *N. benthamiana* (5 to 7 weeks post inoculation) were directly homogenized in 2–5-fold volume of negative stain solution. This consisted of 2% ammonium molybdate, pH 6.5, with one drop of 0.5% bovine serum albumin (BSA) added. Viral particles were adsorbed by floating a pioloform carbon-coated copper grid for 5 min on the crude sap preparation. Finally, grids were rinsed with 5 drops of 2% ammonium molybdate and dried. The preparations were used for size measurements of virions including spikes.

Immunosorbent electron microscopy (ISEM) and immunoelectron microscopy (IEM) decoration experiments targeting the viral nucleocapsid protein were done as described in [27, 28], using the JKI-1607 antiserum to AaNV. Fragments (ca. 2 mm in diameter) of a younger frizzy leaf from systemically infected *N. benthamiana* were embedded in Epon 812 after consecutive fixation of samples with 2.5% glutaraldehyde and 0.5% osmium tetroxide.

Ultrathin sections of 70 nm were prepared with an ultramicrotome UC7 (Leica, Germany) using a DiATOME diamond knife (Switzerland) and were placed on 75 mesh pioloform carbon-coated nickel grids. The grids were stained with 1% uranyl acetate for 30 min and grids were examined in a Tecnai G2 Spirit electron microscope at 80 kV. Images were taken with a 2 K Veleta camera. Brightness and contrast were adjusted when necessary using Adobe Photoshop CS6.

### Purification of rhabdovirus nucleocapsids

Isolation of rhabdovirus nucleocapsids was performed using a modification of a method described by Roggero et al. [29, 30]. Briefly, 100 g infected leaf materials of *N. benthamiana* were blended for 1 min in 500 ml homogenisation buffer consisting of 100 mM Tris-HCl, pH 8, containing 20 mM sodium sulfite, 10 mM Na-DIECA and 5 mM Na-EDTA. The homogenate was filtered through cheesecloth and centrifuged at 3000 rpm for 10 min in a GSA rotor (Sorvall). The supernatant was centrifuged at 25,000 rpm for 30 min in a 45 Ti fixed-angle rotor (Beckman Coulter), and the pellets were resuspended in 180 ml homogenisation buffer plus 2% (w/v) lauryl sulfobetaine and stirred for 1 h at 4 °C, followed by centrifugation at 9000 rpm for 10 min in a GSA rotor (Sorvall). The supernatant was placed onto a 20% sucrose cushion in homogenisation buffer (3.5 ml/tube) and ultracentrifuged at 25,000 rpm for 2.5 h in a SW 28 Ti rotor (Beckman Coulter). Then, the pellets were resuspended in 1 ml 10 mM Tris-HCl, pH 8, and centrifuged at 14,000 rpm in a MiniSpin centrifuge (Eppendorf). The supernatant was then placed onto preformed cesium sulfate-gradients (260, 405 and 575 mg/ml [w/v], respectively) in 10 mM Tris-HCl, pH 8, and ultracentrifuged at 35,000 rpm for 20 h in a SW 55 Ti rotor (Beckman Coulter). Opalescent bands were collected with a peristaltic pump, diluted to 25 ml with 10 mM Tris-HCl, pH 8, and ultracentrifuged at 40,000 rpm for 3 h in a 70 Ti rotor (Beckman Coulter). The resulting pellet was resuspended in 500 ml 10 mM Tris-HCl, pH 8, and used for nucleocapsid quantification by UV spectroscopy, for EM examination and antiserum production.

### Antibody production and serological detection

For production of an antiserum to AaNV (designated JKI-1607), a purified nucleocapsid preparation (approximately 250 μg/ml in 0.01 M Tris-HCl, pH 8.0) was mixed with Freund’s complete adjuvant (1:1) and injected directly into the hindleg muscles (IM) of a cross-bred rabbit. Such injections were repeated two times using Freund’s incomplete adjuvant after 1 week and after 9 weeks. One week after the last injection, the rabbit was bled at weekly intervals for 1 month. Immunoglobulin G (IgG) isolation and conjugate production were performed according to [31]. The specificity of the AaNV IgGs was tested at a dilution of 1:1000 [v/v] in a DAS-ELISA format [31] using extracts from EMDV-, PhCMoV- and AaNV-infected *N. benthamiana*. In reciprocal DAS-ELISA experiments, antisera to EMDV (JKI-1073) and PhCMoV (JKI-2051) were tested against extracts from AaNV-inoculated plants (upper, non-inoculated leaves). DAS-ELISA was also performed to confirm the presence of AaNV in plants inoculated for the (limited) host range study. The calculation of cut-off values for each ELISA plate carried out according to the Technical Information by Bioreba [32].

### Whole genome sequencing

Total RNA (totRNA) was extracted from *N. benthamiana* infected leaf material using innuPREP RNA Mini Kit (Analytik Jena AG, Jena, Germany) following the manufacturer’s protocol. Ribosomal RNA (rRNA) was depleted using RiboMinus Plant kit (Invitrogen, Carlsbad, CA, USA) according the manufacturer’s protocol. Random cDNA was synthesized using ProtoScript II Reverse Transcriptase (New England Biolabs, Beverly, MA, USA) and 8 N random primers. The second-strand was synthesized with NEBNext Ultra II Non-Directional RNA Second Strand Synthesis Module kit (New England Biolabs (NEB), Beverly, MA, USA). A library was prepared using Nextera XT Library kit (Illumina) and subsequently run on a MiSeq v3 platform as pair-end reads (2 × 301).

### Sequencing of 5′ and 3′ ends

To obtain the 5′ and 3′ ends of the full-length AaNV sequence, RNA ligase mediated amplification of cDNA ends (RLM-RACE) [33–35] and RNA poly A tailing were used, respectively.

For the 5′ end, cDNA was produced using a virus specific primer (HZ-454 5′ ACT CTT GGT ACA GCA ACT CGT 3′) located 461 bases from the end. The resulting cDNA was purified using the DNA Clean & Concentrator kit (Zymo Research, Orange, CA, USA). An adaptor (Oligo1rev 5′ PO_4_-GAT CCA CTA GTT CTA GAG CGG C-AminoC3 cordycepin 3′ adapted from [34]) was ligated to the cDNA using T4 RNA ligase 1 (NEB) and the ligated cDNA was purified. PCR amplification of the 5′ end was performed using a primer (Oligo2for 5′ GCC GCT CTA GAA CTA GTG GAT C 3′) complementary to the ligated adaptor and a virus specific primer (HZ-452 5′ TCC ACA AGT TGC AAG CAG GT 3′) 397 bases from the genome end. A band of approximately 400 bases was obtained.

For obtaining the 3′ end, totRNA was poly-A tailed with the A-Plus™ Poly(A) Polymerase Tailing kit (Cellscript, Madison, WI, USA) and cDNA was synthesized using a primer (HZ-413 5′ GGA CAT TGT CCG GAT GGT CT 3′) binding 361 bases from the 3′ end of the RNA. The 3′ end was amplified by PCR using HZ-413 and oligo(d)T primer (5′ CCT CGG GCA GTC CTT TTT TTT TTT TTT TTT T 3′) [36].

The PCR products of both ends were cleaned using the Zymoclean Gel DNA Recovery (Zymo Research) and cloned with NEB PCR Cloning Kit (NEB). Cloning and plasmid amplification were carried out according to the manufacturer’s instructions. Purification of plasmids was carried out using the NucleoSpin Plasmid EasyPure Kit (Macherey-Nagel, Düren, Germany); sequencing (ten colonies in both directions) was carried out at Macrogen (Seoul, Korea) and Eurofins Genomics (Ebersberg, Germany).

### Sequence analysis

The reads produced from the MiSeq platform were analysed with Geneious software (v 11.0.4) (Biomatters Limited, Auckland, New Zealand). The raw reads were quality trimmed (error limit = 0.05), size filtered > 99 nt, error corrected and normalised using BBNorm (v. 37.64) tool, followed by de novo assembly with Geneious assembler. Assembled contigs were then used to search for similar sequences by BLASTn and BLASTx using the National Centre for Biotechnology Information (NCBI) GenBank non-redundant nucleotide and protein databases, respectively. Mapping of the clean reads to the complete viral genome sequence as a reference was performed using the mapping to reference tool in Geneious. Open reading frames were identified by Find ORF tool and were used to find similar sequences and conserved domains in BLASTp.

Sequence alignments were done with clustalW and phylogenetic trees (Neighbour-Joining algorithm, 1000 bootstrap replications) were created using MEGA 7.0.26 [37, 38]. The full genome of the virus was submitted to GenBank using Sequin application (v 15.50). Importin-dependent nuclear localisation signals were predicted using cNLS Mapper [39] and nuclear export signals (NES) were predicted using NetNES 1.1 [40].

### Reverse transcription polymerase chain reaction (RT-PCR) for detection and confirmation

Two primers (HZ-408 5′ GCA CGA TAA AGG CTG CAT CG 3′ and HZ-409 5′ TTG TGC ATC CTC TGT CGG AC 3′) were designed (Geneious design new primer tool) to confirm the virus presence by RT-PCR. The primers were designed to amplify a 971 bp fragment of the RNA-dependent RNA polymerase gene.

Extraction of totRNA was done from leaf tissues as described above, and cDNA was produced using HZ-409 primer. The cDNA product was used for PCR using OneTaq DNA Polymerase kit (NEB) (35 cycles of 30 s at 94 °C, 45 s at 52 °C, 1 min at 68 °C and a final elongation step for 4 min at 68 °C). The amplified PCR products were subject to electrophoresis on a 1.0% (w/v) agarose gel stained with ethidium bromide. The specificity of the designed primers was confirmed by testing EMDV- and PhCMoV-infected plants.

### Infectivity assays

AaNV-infected *N. benthamiana* leaves were used to inoculate *N. benthamiana*, *M. sativa*, *M. lupulina*, *Pisum sativum* and *Vicia faba* mechanically. Briefly, symptomatic leaves were homogenized in Norit inoculation buffer (50 mM phosphate buffer, pH 7, containing 1 mM ethylenediaminetetraacetic acid (Na-EDTA), 20 mM sodium diethyldithiocarbamic acid (Na-DIECA), 5 mM thioglycolic acid, 0.75% activated charcoal and 30 mg Celite). Using a glass spatula, the homogenate was gently rubbed onto the leaves which were then rinsed with water. The inoculated plants were kept under greenhouse conditions (at 22 °C; photoperiod of 16 h light [natural daylight with additional growth light Phillips IP65, 400 W] and 8 h dark) and regularly inspected for symptoms for at least three weeks after inoculation.

## Results

### Virus transmission and maintenance

Upon receiving the infected alfalfa sample, the virus was mechanically inoculated onto standard indicator plants including *N. benthamiana* which were inspected for symptoms twice weekly. In *N. benthamiana*, chlorotic lesions appeared on inoculated leaves followed by systemic leaf rolling, mottling and yellowing in week three or four post inoculation. The virus was maintained continuously on *N. benthamiana* by regular mechanical passage onto young seedlings.

### Virus morphology and cellular localisation

**Fig. 1:**
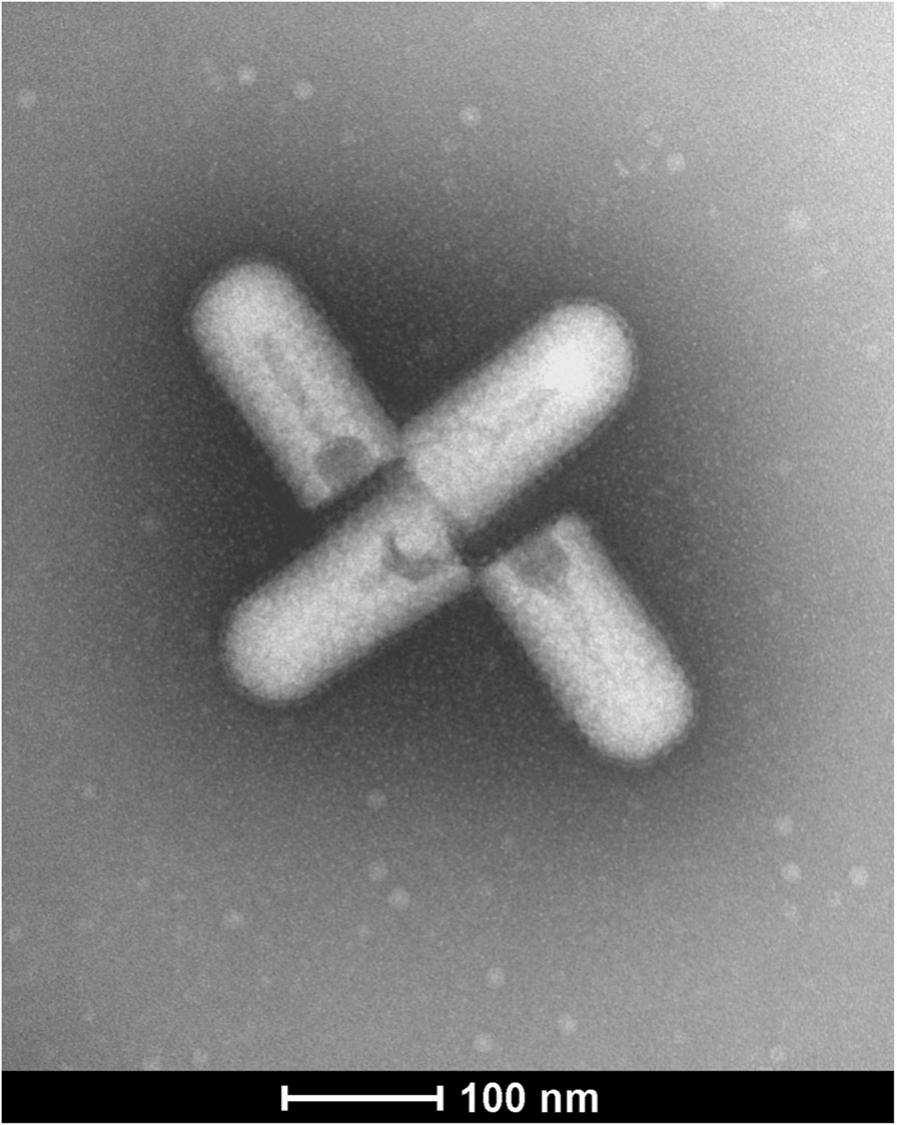
Electron micrograph of crude plant sap preparations of AaNV-infected *N. benthamiana* leaves. Four shorter mature bacilliform virions with average sizes of 167 nm in length and 86 nm in diameter

To elucidate the aetiology of the alfalfa disease, transmission electron microscopy (TEM) was performed on infected *N. benthamiana* plants following mechanical inoculation. Bacilliform-shaped virus particles were observed (Fig. 1). Using ammonium molybdate instead of uranyl acetate as negative stain was less disruptive on particle appearance. Only few mature virions displaying various degrees of disruption were detected in adsorption preparates. Preliminary measurements obtained from *n* = 40 revealed virion sizes ranging from 180 to 200 nm in length and 85–95 nm in diameter. The outer surface of virions is preserved comprising the lipid bilayer carrying the spikes, likely glycoproteins. Virions shown in Fig. 1 are less disrupted with matrix proteins and envelope mostly intact. About 15% of the measured particles like those depicted in Fig. 1 were of shorter size (average length 167 nm) and may indicate defective particles not comprising the complete viral genome.

**Fig. 2:**
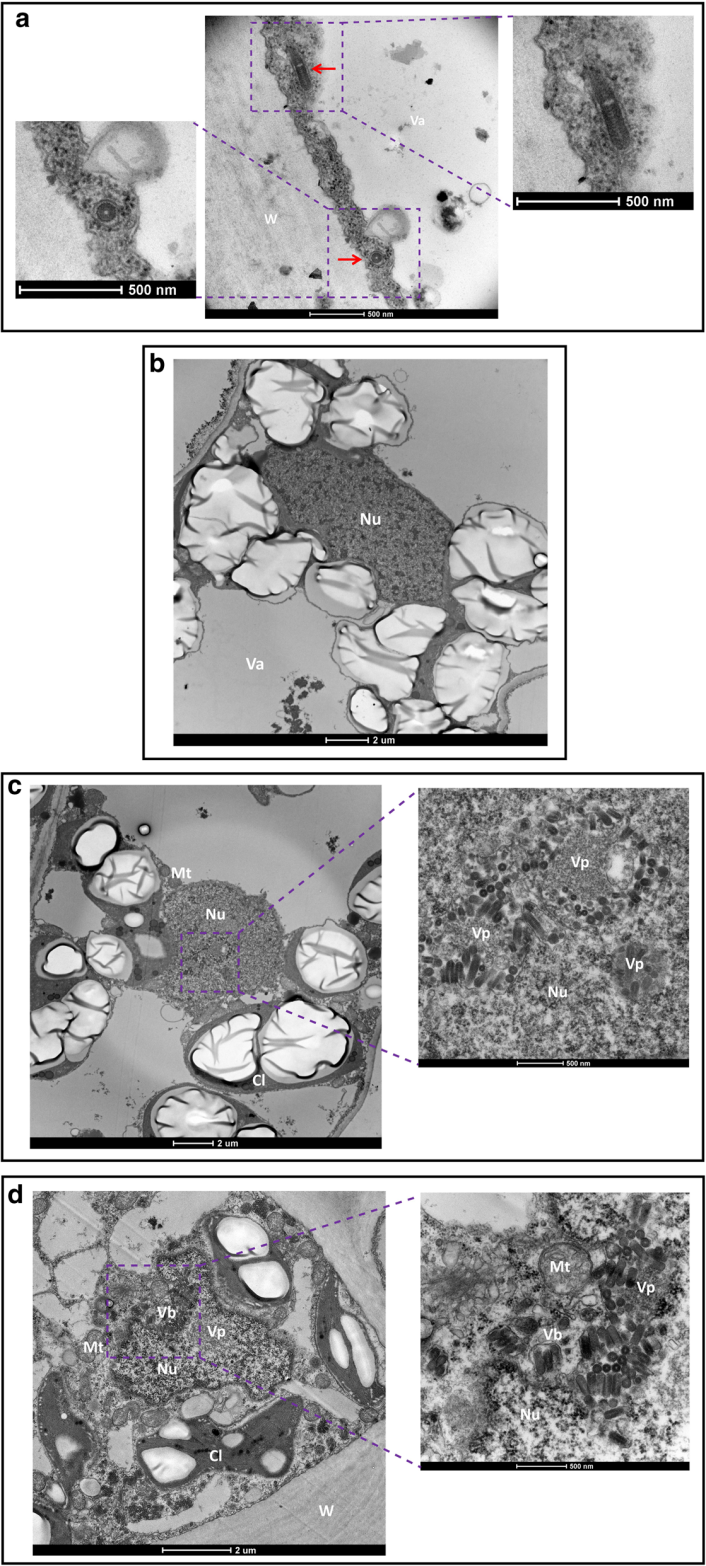
Electron micrographs of thin sections of AaNV-infected *N. benthamiana* cells. **a** Arrows indicate transversely (bottom) and longitudinally (upper part) cut particles in the cytoplasm located between the cell wall (W) and the vacuole (Va) of an epidermal cell; **b** Non-infected nucleus with heterochromatin and homogenous nuclear matrix; **c** Electron-dense granular areas throughout the nucleus are thought to represent viroplasm (Vp) with virions placed next to it; **d** arrays of mature virions budding (Vb) into the perinuclear space surrounded by the nuclear membrane. The cell wall (W), chloroplast with starch granules (Cl), nucleus (Nu), mitochondrion (Mt), vacuole (Va), virus budding (Vb) and viroplasm (Vp) are indicated

When ultrathin sections of embedded symptomatic *N. benthamiana* leaf tissue were analysed, very few virus particles were found in the cytoplasm only. Figure 2a shows two virus particles in epidermal cells. The transversely cut particle seems to be complete with attachment of glycoproteins visible (lower arrow, left hand side). Figure 2a (upper arrow, right hand side) seems to show two longitudinally particles appearing blunt end to blunt end and thus looking like a larger particle. Both epidermal and mesophyl cells were infected.

In infected cells, the shape of the nucleus can change to a more condensed circular or even a distorted shape (Fig. 2c-d) compared to the ellipsoidal form present in healthy cells (Fig. 2b). In heavily infected cells, not only the nuclear compartments were affected but also chloroplast were deformed (Fig. 2d). In the nuclei, granular areas distinct from heterochromatin were found representing putative virus replication sites known as viroplasms (Vp). Adjacent to them virus particles could be found (Fig. 2c and d). In Fig. 2c, there are few virions around the Vp and no virions were observed in the cytoplasm. In addition, vesicles or virus buddings (Vb) containing one or more complete viruses were visualized around the nucleus and in the cytoplasm of infected cells (Fig. 2d). Figure 2d also shows virus particles budding from the inner nuclear envelope in the perinuclear space.

### Sequence analysis

A total of 1,561,664 reads were generated from the MiSeq sequencing. After quality trimming and size filtering, 1,141,662 quality-filtered reads were used for normalisation and de novo assembly. From the 23,180 assembled contigs, a contig of 13,854 nucleotides showed 66.9% identity (7% coverage and 3e-50 E-value) to black currant-associated rhabdovirus 1 (BCaRV-1) (MF543022), 66.2% (6% coverage and 2e-45 E-value) to datura yellow vein virus (DYVV) (NC_028231) and 66.2% (9% coverage and 5e-41 E-value) to sonchus yellow net virus (SYNV) (NC_001615) using BLASTn. Using BLASTx, the contig shared 44.9% (34% coverage and zero E-value) identity to DYVV (YP_009176977), 43.62% identity (35% coverage and zero E-value) to SYNV (NP_042286) and 43.5% (34% coverage and zero E-value) to BCaRV-1 (AUW36419). To determine the 5′ and 3’ends, primers were designed to anneal close to the assembled contig ends. The sequences of the two ends were assembled to the contig and the full-length genome sequence was determined as 13,875 bases in length with 29,727 mapped reads, 40.6% G + C content and mean coverage of 586X (GenBank accession number MG948563). The sequencing dataset generated in this study is available from the corresponding author upon request.

**Fig. 3:**
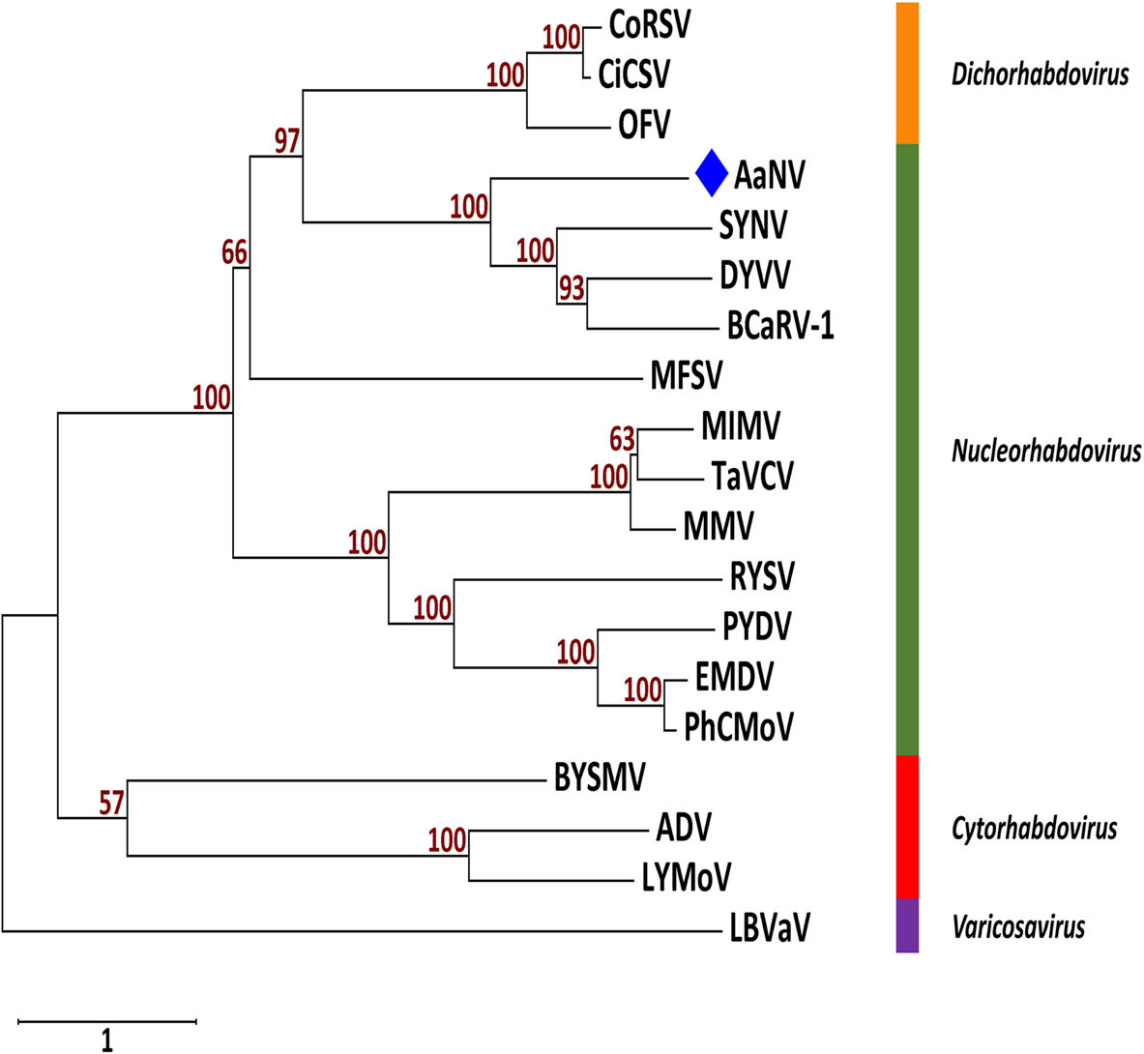
Unrooted neighbour-joining phylogenetic tree [Genetic distance model (Jones-Taylor-Thornton (JTT) model) and 1000 bootstrap replications] based on the amino acid sequence alignment of the L protein of selected members of different genera of the family *Rhabdoviridae*. AaNV indicated by a blue solid diamond shape. The bootstrap values above 50% are indicated for each node. The names and the accession numbers of the viruses are as follow: *Nucleorhabdovirus* (green): alfalfa-associated nucleorhabdovirus (AaNV; QAB45076), black currant-associated rhabdovirus 1 (BCaRV-1; AUW36419), datura yellow vein virus (DYVV; YP_009176977), eggplant mottled dwarf virus (EMDV; YP_009094358), maize fine streak virus (MFSV; YP_052849), maize Iranian mosaic virus (MIMV; YP_009444713), maize mosaic virus (MMV; YP_052855), physostegia chlorotic mottle virus (PhCMoV; ARU77002), potato yellow dwarf virus (PYDV; YP_004927971), rice yellow stunt virus (RYSV; NP_620502), sonchus yellow net virus (SYNV; NP_042286) and taro vein chlorosis virus (TaVCV; YP_224083). *Cytorhabdovirus* (red): alfalfa dwarf virus (ADV; YP_009177021), barley yellow striate mosaic virus (BYSMV; YP_009177231) and lettuce yellow mottle virus (LYMoV; YP_002308376). *Dichorhabdovirus* (orange): citrus chlorotic spot virus (CiCSV; ARJ35804), coffee ringspot virus (CoRSV; YP_009507905), orchid fleck virus (OFV; YP_001294929). *Varicosavirus* (violet): lettuce big-vein associated virus (LBVaV; YP_002308576)

A pairwise nucleotide sequence alignment of the novel genome to selected rhabdoviruses and a phylogenetic tree was generated. ClustalW pairwise analysis showed that the AaNV sequence has 39.8% nt identity to BCaRV-1, 38.8% to DYVV and SYNV (Additional file 1: Figure S1a). Moreover, the AaNV sequence falls within the genus *Nucleorhabdovirus* in a clade with SYNV, BCaRV-1 and DYVV (Additional file 1: Figure S1b). This clustering was supported by a neighbour joining tree of the L protein amino acid sequences of selected members of the family *Rhabdoviridae* (Fig. 3).

### The genome organisation of AaNV

**Fig. 4:**
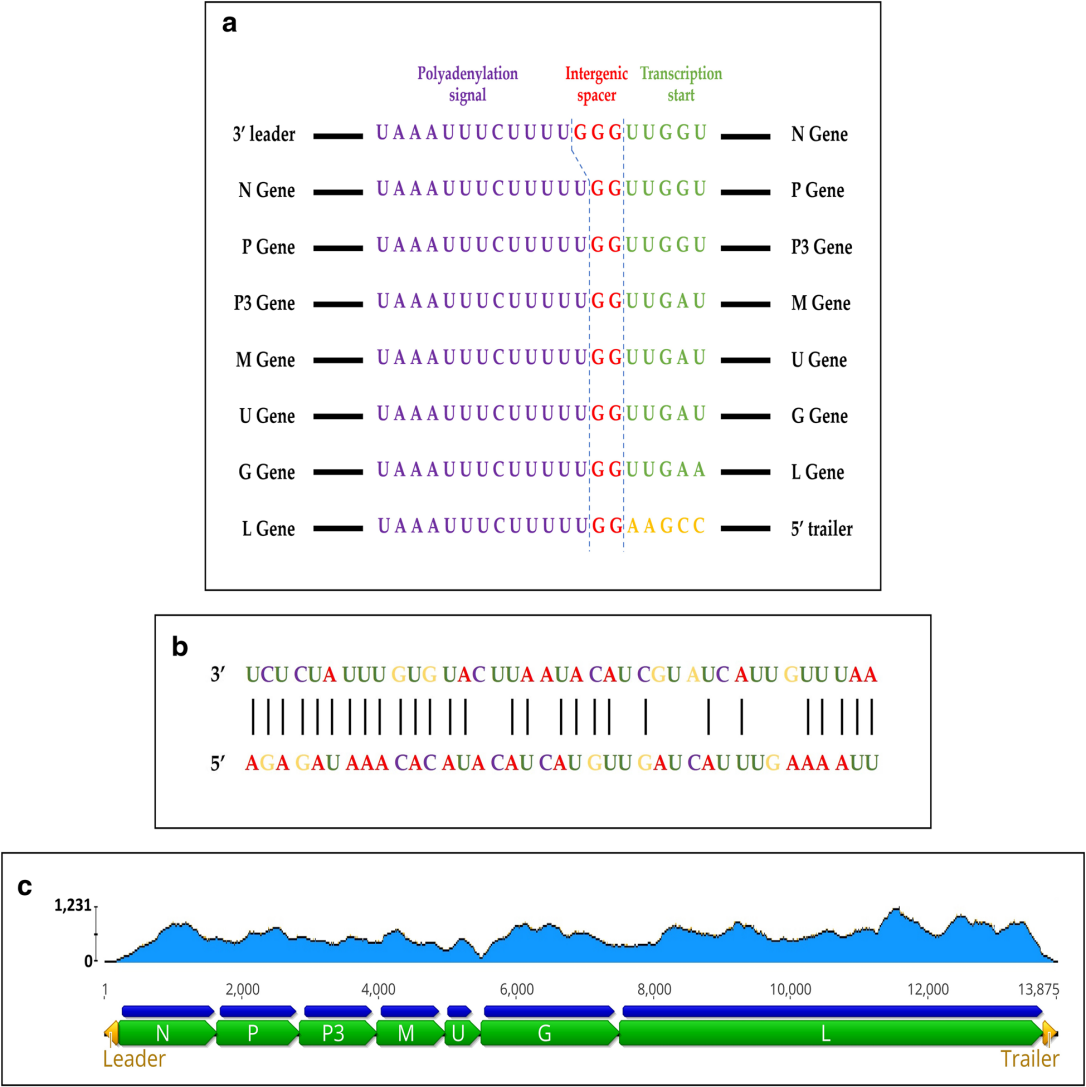
**a** The intergenic regions of the alfalfa-associated nucleorhabdovirus (AaNV) genome; the polyadenylation signal, the intergenic spacer and the transcription start site; **b** Alignment of ends of AaNV 3′ leader (l) and 5′ trailer (t) sequences (complementary nucleotides are indicated by vertical black lines); **c** Schematic representation of the full sequence of AaNV and its genome organisation. The open reading frames N, P, P3, M, U, G, L with their CDS are indicated as green and blue block arrows, respectively. The yellow block arrows represent the 3′ leader (l) and the 5′ trailer (t). The read map distribution is shown in light blue over the genome

Six putative open reading frames (ORFs) were identified in the antigenomic sense based on the genome organisation described for other nucleorhabdoviruses; nucleocapsid (N), phosphoprotein (P), putative cell to cell movement protein (P3), matrix protein (M), glycoprotein (G) and RNA-dependent RNA polymerase (L). Highly conserved regulatory regions separating the genes were identified. At the junctions between the genes, the consensus motif is (3′ UAA AUU CUU UUU GGU UG 5′), which slightly differs between the 3′ leader and N gene, and between the L gene end and the 5′ trailer (Fig. 4a). The presence of a seventh ORF with unknown function (U), between M and G was identified as it is flanked by the intergenic region consensus motif. Moreover, the 3′ leader (l) and the 5′ trailer (t) have complementary sequences of 43.1% nt identity (Fig. 4b). Therefore, the complete genome organisation was determined as 3′ l–N–P–P3–M–U–G–L–t 5′ (Fig. 4c). Additionally, comparisons between the consensus sequence of the intergenic conserved sequences, the 3′ and the 5′ ends, and the genome organisation of AaNV and selective members of the *Nucleorhabdovirus* genus are shown in Additional file 1: Figure S2.

### Predicted protein features in silico

The AaNV protein sizes range from 113 amino acid (aa) for the U protein to 2038 aa for the L protein with molecular masses of 12.4 kDa and 234.8 kDa, respectively (Table 1). The N and G proteins have neutral isoelectric points (IEP) of 7.1 and 7.3. U and P are acidic proteins while P3, M and L are basic proteins (Table 1). Comparing the protein sequences of AaNV with those of BCaRV-1 and DYVV showed that the predicted proteins of AaNV are more closely related to those of DYVV except for the glycoprotein, which is more closely related to that of BCaRV-1 (Table 1). The aa sequences identities were between 11.5 and 35.8% compared to BCaRV-1 and between 14 and 33.7% for DYVV (Table 1). Additionally, the nuclear localisation signals (NLS, or a karyophilic domain) and the nuclear export signals (NES) of the proteins were predicted (Table 1). The highest cNLS mapper scores were for N, P and L (12.7, 10 and 10, respectively), followed by P3 and G with scores of 7 and 6. The M protein had the lowest score with 4.3 while the U ORF did not score any NLS. The cNLS scores predicted an exclusive nuclear localisation for N, P and L proteins, a partial nuclear localisation for P3 and G proteins, and a nuclear and cytoplasmic localisation for M protein (Table 1). Moreover, four of these proteins have a detectable NES (Table 1).

**Table 1:** Characteristics of alfalfa-associated nucleorhabdovirus (AaNV) encoded proteins [sizes in amino acids (aa), molecular masses (MW) in kilo Dalton (kDa), the isoelectric points (IEP), predicted cell nuclear localisation signals (cNLS) and nuclear export signals (NES)]

Putative gene function	Gene	Size (aa)	MW (kDa)	pairwise aa sequence identity (%)		IEP	Predicted NLS					NES position
				BCaRV-1	DYVV		Position	Partite	aa sequence	cNLS Mapper Score	Predicted location	
Nucleocapsid protein	N	443	50.3	33.5	36.6	7.1	408	Bi^c^	RAGIKRQAGDHETQGTKRARTS	12.7	eN	351
Phosphoprotein	P	363	41.1	11.5	14	6.4	146	Mono^d^	RGNKRRKRSD	10	eN	ND
Putative cell-to-cell movement protein	P3	322	37.2	14.6	22.4	8.6	17	Bi	PTKKRTSQDKYNFRSTESLYAEPYNKIIRTK	7	pN	ND
Matrix protein	M	277	31.4	14.9	16.5	7.6	213	Bi	RSSVKITGKQMRARSSSRSRSPYKVSLSSNKRTYLD	4.3	N/Cp	136
Unknown protein	U	113	12.4	NA^a^	NA	4.0	ND^b^	ND	ND	ND	NA	31
Glycoprotein	G	629	71.6	26.4	24.6	7.3	443	Bi	KSAYKKKLPYEVTAWNGDKIMSEYPYKNIVVE	6	pN	ND
RNA-dependent RNA polymerase	L	2038	234.8	35.8	36.7	7.8	780	Bi	EKTAIKRRMRAFRDDLGQKMKKR	10	eN	88,90 and 149

The amino acid sequence identities between the putative gene products of AaNV and those of black currant-associated rhabdovirus 1 (BCaRV-1; MF543022) and datura yellow vein virus (DYVV; NC_028231). The predicted protein cell nuclear localisation signal (cNLS; http://nls-mapper.iab.keio.ac.jp/cgi-bin/NLS_Mapper_form.cgi) Mapper score and Nuclear export signals (NES) are also mentioned. The cut-off values of the cNLS mapper scores: 8, 9, or 10 = the protein is predicted to be exclusively localised to the nucleus (eN), 6, 7 or 8 = partially localised to the nucleus (pN), 3, 4, or 5 = localised to both the nucleus and the cytoplasm (N/Cp), and 1 or 2 = localised to the cytoplasm (Cp). ^a^NA: not applicable. ^b^ND: not detectable. ^c^Bi: Predicted bipartite NLS^d^ Mono: Predicted monopartite NLS

### Antiserum production, serological specificity and diagnostic RT-PCR

The specificity of the AaNV antiserum was confirmed by DAS-ELISA using plant material infected with either AaNV or two other rhabdoviruses (EMDV and PhCMoV). The AaNV antibodies reacted only with plant material infected with AaNV but neither with non-inoculated nor non-infected plant material nor with plants infected with EMDV or PhCMoV (Table 1). In reciprocal tests, antisera to EMDV and PhCMoV did not react with AaNV-infected plant tissue. Diagnostic primers were designed to confirm the presence of AaNV by RT-PCR resulting in a 971 bp amplicon. The primers were specific to the partial sequence of the L-ORF of AaNV and did not amplify other nucleorhabdoviruses tested, i.e., EMDV and PhCMoV.

The specific recognition of nucleocapsids by the AaNV antibodies were demonstrated using IEM. Only nucleocapsid structures reacted with antibodies but not complete virions, see Additional file 1: Figure S3a displaying enriched but undecorated nucleocapsids from the crude sap samples after the preincubation with antiserum (immunosorbent step), and Additional file 1: Figure S3b showing nucleocapsids covered with antibodies after the decoration step. With antiserum against EMDV, neither enrichment nor decoration of nucleocapsids were obtained using IEM (data not shown).

### Infectivity tests

**Fig. 5:**
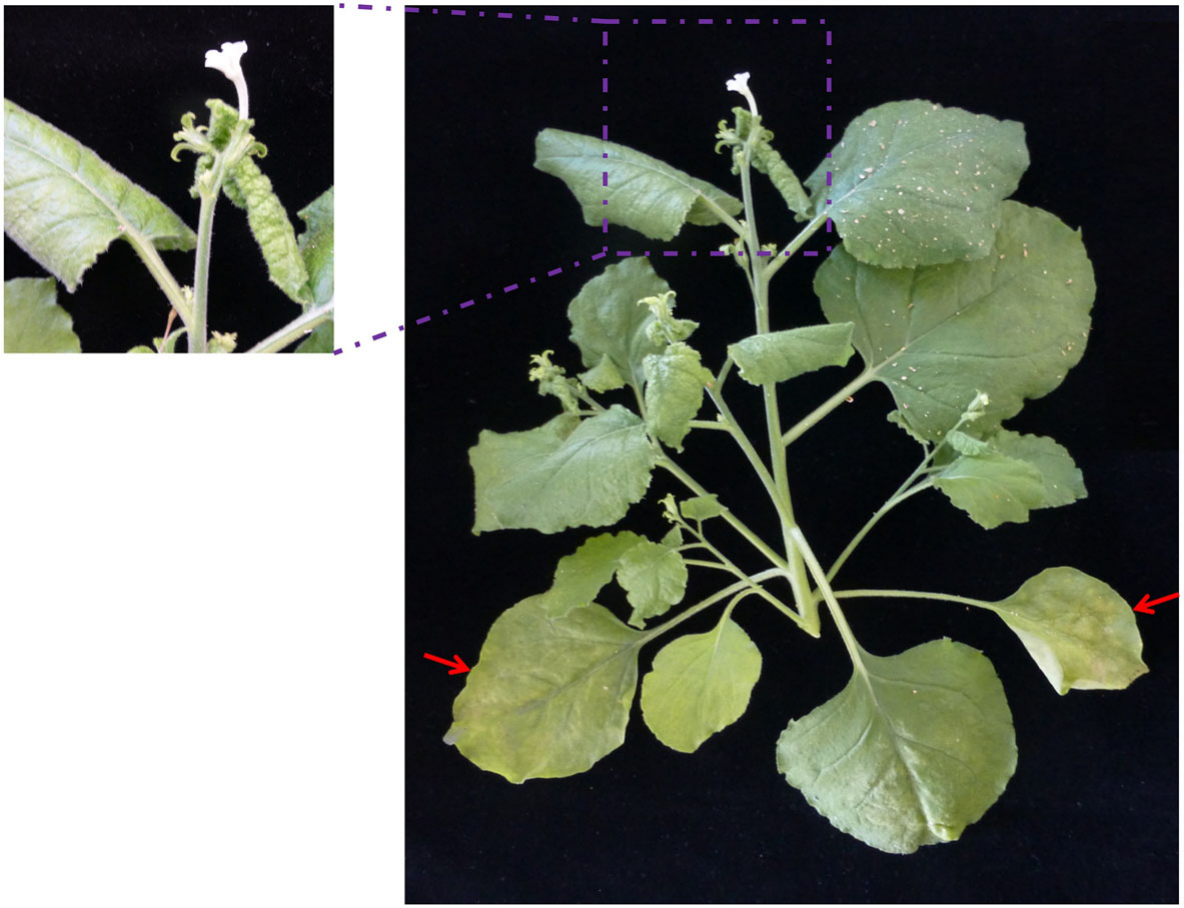
Photo of AaNV-infected *N. benthamiana* plant. The plant shows systemic leaf rolling, mottling, yellowing and curling, and chlorotic lesions on inoculated leaves at approximately 4 weeks post inoculation. Red arrows indicating inoculated leave

In a limited host range study, the virus was successfully transmitted to *N. benthamiana*, *P. sativum*, and *V. faba*. Mechanically inoculated *N. benthamiana* plants showed systemic infection. Systemic symptoms consisted of leaf mottling, yellowing and curling at approximately 4 weeks after inoculation (Fig. 5). However, inoculated *P. sativum* and *V. faba* showed either no symptoms or a slight leaf mottling and the infection rate was low on these hosts (only 4 out of 36 *V. faba* and 1 out of 30 *P. sativum* plants). To confirm the infections, DAS-ELISA and RT-PCR were performed. Use of the AaNV antiserum in DAS-ELISA confirmed AaNV infections at high titres in *N. benthamiana* and at lower titres in *P. sativum* and *V. faba* and the absence of detectable virus from AaNV-inoculated *M. lupulina*, *M. sativa*, *T. pratense*, *T. repens* and *C. quinoa* (Table 2). Infections were also confirmed by RT-PCR. It was not possible to transmit AaNV mechanically to *M. lupulina* and *M. sativa* (21 and 18 plants tested, respectively). Plants remained symptomless and all the samples tested negative in DAS-ELISA and RT-PCR. Additionally, mechanical inoculation using fresh material from AaNV-infected *V. faba* and *P. sativum* plants failed to induce infection in *V. faba*, *P. sativum*, *M. lupulina* and *M. sativa*.

**Table 2:** DAS-ELISA reactions of various antisera raised against different plant nucleorhabdoviruses and limited host range study

Host species	Inoculated virus	Antisera		
		AaNV (JKI-1607)	EMDV (JKI-1073)	PhCMoV (JKI-2051)
*N. benthamiana*	AaNV	+++^a^	–	–
*P. sativum*	AaNV	+	NT ^b^	NT
*V. faba*	AaNV	+	NT	NT
*M. sativa*	AaNV	–	NT	NT
*M. lupulina*	AaNV	–	NT	NT
*T. repens*	AaNV	–	NT	NT
*T. pratense*	AaNV	–	NT	NT
*C. quinoa*	AaNV	–	NT	NT
*N. benthamiana*	EMDV	–	+++	–
*N. benthamiana*	PhCMoV	–	–	+++
Buffer		–	–	–
*N. benthamiana*	Non-inoculated	–	–	–
*P. sativum*	Non-inoculated	–	NT	NT
*V. faba*	Non-inoculated	–	NT	NT
*M. sativa*	Non-inoculated	–	NT	NT

^a^Following a substrate incubation for 1 h, DAS-ELISA reactions were classed as follows: negative reaction (−): ≤ cut-off point (= OD_A405_ 0.025); weak reaction (+): cut-off point to 1.0, intermediate reaction (++): 1.0 to 2.0, strong reaction (+++): > 2.0). ^b^NT = not tested

## Discussion

Using EM and HTS technologies, the presence of a novel nucleorhabdovirus in alfalfa was established. The bacilliform appearance of the viral particles observed in infected *N. benthamiana* tissues is consistent with observations on previously described plant rhabdoviruses. Preliminary measurements indicated particle sizes within the range of the known nucleorhabdoviruses (130 to 300 nm × 45 to 100 nm in diameter [41]). The virions of AaNV had an average length of 180–200 nm and measured 85–95 nm in diameter. The observed ultra-cellular deformations of nuclei and chloroplast in epidermis and mesophyl cells are in accordance with the distorted phenotype of systemically infected *N. benthamiana* plants showing leaf rolling, mottling and yellowing.

The species demarcation criteria for the genus *Nucleorhabdovirus* state that a new species should have three characteristics [17]; a new species should have a minimum nucleotide divergence of 50% in cognate genes, can be clearly distinguished in serological tests or by nucleic acid hybridisation, and should occupy a different ecological niche (differences in hosts and/or vectors). The AaNV genome shares 39.8% nucleotide identity with BCaRV-1, its closest relative in the genus *Nucleorhabdovirus*. Moreover, all its ORFs have less than 40% amino acid sequence identity with their most closely related sequences in other rhabdoviruses. In addition, the AaNV antiserum reacted specifically with AaNV-infected plant tissue while antisera to EMDV and PhCMoV, two other nucleorhabdoviruses, failed to react with AaNV infected plant tissues in DAS-ELISA. Furthermore, the primers for RT-PCR are specific for AaNV. As for the third demarcation criterium, AaNV was originally identified in *Medicago sativa*, an important legume crop. However, the mode of transmission and/or potential vectors have not yet been identified. As a consequence, AaNV should be considered as a new virus species in the *Nucleorhabdovirus* genus.

As with all rhabdoviruses, the genome of AaNV has highly conserved regulatory regions (intergenic regions) separating its ORFs and complementary 3′ leader and 5′ trailer sequences. The intergenic regions of AaNV are closely related to those of DYVV, BCaRV-1 and SYNV [25, 42]. The predicted features of AaNV proteins are similar to those of related nucleorhabdoviruses. The individual proteins of AaNV are similar in size to their homologs in DYVV and BCaRV-1. The predicted isoelectric point (IEP) of N protein of AaNV is the same as that of DYVV [42]. Similar to DYVV, P3, M and L are basic proteins and P is an acidic protein.

The only difference is the G protein which is neutral in case of AaNV and acidic for DYVV. In addition to the six main nucleorhabdovirus proteins (N, P, P3, M, G and L), a new ORF (U) with unknown function was identified. Its predicted protein has an acidic IEP. All the seven transcription units and the leader are predicted to be polyadenylated, but its functionality still needs to be proven.

Nucleorhabdoviruses are known to establish virus replication factories in the nuclei of infected plant cells [21]. All AaNV proteins except U, display predicted mono- or bipartite nuclear localisation sequences (NLS) suggesting their independent importation into the nucleus. The presence of both the NLSs and the leucine-rich nuclear export signals (NESs) in N, M and L proteins indicates the ability of these proteins to shuttle between the nucleus and the cytoplasm through coordination of these transport signals. Although the unknown protein (U) seems to lack a NLS, the observation that it has an NES suggests its ability to be exported out of the nucleus.

AaNV was mechanically transmitted to *N. benthaminana*, *P. sativum* and *V. faba*. Although it did not show any noticeable or only slight mottling symptoms on *P. sativum* and *V. faba*, low infection rates were confirmed by DAS-ELISA. Interestingly, the virus could not be mechanically transmitted to *M. sativa* nor *M. lupulina*. It is not known if this is due to the serial passaging on *N. benthamiana* for propagation purposes and therefore a host adaption effect. The biological impact of the observed smaller sized particles of 167 nm length for mechanical transmission and host interactions awaits further investigation. As a (insect) vector has not been identified yet, it is unclear how the transmission from *M. sativa* to *M. sativa* would occur naturally or if *P. sativum* and *V. faba* crops or some weed species could act as natural alternative reservoirs for AaNV. It is also unknown if this virus still occurs naturally in alfalfa in the area it was originally found, or elsewhere in Europe. As no sequence data nor serological data are available for LEV, it is unclear whether these “historic” findings are related to AaNV.

## Conclusions

In the present study, we describe a novel nucleorhabdovirus originating from infected *M. sativa* from Austria. Using HTS, we were able to determine the full-length sequence of this virus which was tentatively named AaNV. Since the sequence identity to BCaRV-1, its closest known relative, was only 39.8%, AaNV represents a new species according to the species demarcation criteria set by the International Committee on Taxonomy of Viruses (ICTV) [17]. The site of virus maturation was observed by EM in the nucleus of infected cells thus confirming the phylogenetic assignment. It was possible to transfer AaNV experimentally using mechanical inoculation to *N. benthaminana* as well as other members of the *Fabaceae* family, i.e., *P. sativum* and *V. faba*. Along with ADV and LEV, this is the third rhabdovirus and the second nucleorhabdovirus known to infect *M. sativa* in nature. However, it was not possible to transfer AaNV back to alfalfa by mechanical inoculation. Thus, further research is needed to identify natural vectors of this virus as well as other alternative host plants. The serological and molecular biological assays developed may aid larger surveys addressing these questions.

## Additional file

Additional file 1: **Figure S1.** **a** Pairwise identity matrix of the whole genome sequences of AaNV with selected members of the family *Rhabdoviridae* (ClustalW 2.1); **b** Unrooted neighbour-joining phylogenetic tree [Genetic distance model (Jukes-Cantor) and 1000 bootstrap replications] based on the nucleotide alignment of the whole genomes of AaNV and selected members of different genera of the family *Rhabdoviridae*. AaNV indicated by a blue solid diamond shape. The names and the accession numbers of the viruses are as follow: *Nucleorhabdovirus* (green): alfalfa-associated nucleorhabdovirus (AaNV; MG948563), black currant-associated rhabdovirus 1 (BCaRV-1; MF543022), datura yellow vein virus (DYVV; NC_028231), eggplant mottled dwarf virus (EMDV; NC_025389), maize fine streak virus (MFSV; NC_005974), maize Iranian mosaic virus (MIMV; NC_036390), maize mosaic virus (MMV; NC_005975), physostegia chlorotic mottle virus (PhCMoV; KY859866), potato yellow dwarf virus (PYDV; NC_016136), rice yellow stunt virus (RYSV; NC_003746), sonchus yellow net virus (SYNV; NC_001615) and taro vein chlorosis virus (TaVCV; NC_006942). *Cytorhabdovirus* (red): alfalfa dwarf virus (ADV; NC_028237), barley yellow striate mosaic virus (BYSMV; NC_028244) and lettuce yellow mottle virus (LYMoV; NC_011532). *Lyssavirus* (black): rabies virus (RV; NC_001542). *Perhabdovirus* (violet): eel virus European X (EVEX; NC_022581). **Figure S2.** Comparisons between AaNV and selective members of the *Nucleorhabdovirus* genus. The consensus sequence of the intergenic conserved sequences **(a)**, the 3′ and 5′ ends **(b)**, and the genome organisation **(c)**. The names and the accession numbers of the viruses can be found under Figure S1. **Figure S3.** Electron micrograph of the JKI-1607 reacting with AaNV ribonucleoprotein (RNP). **a** Enriched nucleocapsids after immunosorbent step; **b** Enriched nucleocapsids but not virions are covered (decorated) with antibodies (DOCX 1320 kb)

## Abbreviations

°C: degree Celsius
aa: amino acid
AaNV: alfalfa-associated nucleorhabdovirus
ADV: alfalfa dwarf virus
BCaRV-1: black currant-associated rhabdovirus 1
Bi: predicted bipartite nuclear localisation signals
bp: base pair
BSA: bovine serum albumin
BYSMV: barley yellow striate mosaic virus
cDNA: complementary deoxyribonucleic acid
CDS: coding sequence
CiCSV: citrus chlorotic spot virus
Cl: chloroplast
cNLS: cell nuclear localisation signal
CoRSV: coffee ringspot virus
DAS-ELISA: double antibody sandwich enzyme-linked immunosorbent assay
DYVV: datura yellow vein virus
EM: electron microscopy
EMDV: eggplant mottled dwarf virus
EVEX: eel virus European X
G + C: guanine+cytosine
G: glycoprotein
g: gram
h: hour
HTS: high throughput sequencing
ICTV: International Committee on Taxonomy of Viruses
IEP: isoelectric points
IgG: immunoglobulin G
IM: intramuscular
JKI ID: Julius Kühn Institute identification number
JKI: Julius Kühn Institute
kb: kilobase
kDa: kilo Dalton
kV: kilovoltage
l: 3′ leader
L: RNA-dependent RNA polymerase
LBVaV: lettuce big-vein associated virus
LEV: lucerne enation virus
LYMoV: lettuce yellow mottle virus
M: matrix protein
m: mean
MFSV: maize fine streak virus
mg: milligram
MIMV: maize Iranian mosaic virus
min: minute
ml: milliliter
mm: millimeter
mM: millimolar
MMV: maize mosaic virus
Mono: predicted monopartite nuclear localisation signals
Mt.: mitochondrion
MW: molecular weight
N: Nucleocapsid protein
NA: not applicable
Na-DIECA: sodium diethyldithiocarbamic acid
Na-EDTA: ethylenediamine tetraacetic acid disodium salt dihydrate
NCBI: National Center for Biotechnology Information
ND: not detectable
NES: nuclear export signals
NLS: nuclear localisation signals
nm: nanometer
NT: not tested
nt: nucleotide
Nu: nucleus
OD: optical density
OFV: orchid fleck virus
ORF: open reading frame
P: phosphoprotein
P3: putative cell-to-cell movement protein
pH: power of Hydrogen
PhCMoV: physostegia chlorotic mottle virus
PYDV: potato yellow dwarf virus
RLM-RACE: RNA ligase mediated amplification of cDNA ends
RNA: ribonucleic acid
RNP: ribonucleoprotein
rpm: revolutions per minute
rRNA: ribosomal ribonucleic acid
RV: rabies virus
RYSV: rice yellow stunt virus
sd: standard deviation
sec: second
ssRNA: negative-sense, single-stranded ribonucleic acid
SYNV: sonchus yellow net virus
t: 5′ trailer
TaVCV: taro vein chlorosis virus
totRNA: total ribonucleic acid
Tris-HCl: trisaminomethane-hydrochloride
U: unknown protein
V: virus buddings
v/v: volume/volume
Va: vacuole
Vb: virus budding
Vp: viroplasms
vRNP: viral ribonucleoprotein
W: cell wall
w/v: weight/volume
