# Supplementary material for: Characterisation of a novel nucleorhabdovirus infecting alfalfa (Medicago sativa)
Source: Virol J. 2019 Apr 29;16:55. doi: 10.1186/s12985-019-1147-3 (PMC6489223; doi:10.1186/s12985-019-1147-3)
Supplement: Supplementary file 1 — Figure S1. a Pairwise identity matrix of the whole genome sequences of AaNV with selected members of the family Rhabdoviridae (ClustalW 2.1); b Unrooted neighbour-joining phylogenetic tree [Genetic distance model (Jukes-Cantor) and 1000 bootstrap replications] based on the nucleotide alignment of the whole genomes of AaNV and selected members of different genera of the family Rhabdoviridae. AaNV indicated by a blue solid diamond shape. The names and the accession numbers of the viruses are as follow: Nucleorhabdovirus (green): alfalfa-associated nucleorhabdovirus (AaNV; MG948563), black currant-associated rhabdovirus 1 (BCaRV-1; MF543022), datura yellow vein virus (DYVV; NC_028231), eggplant mottled dwarf virus (EMDV; NC_025389), maize fine streak virus (MFSV; NC_005974), maize Iranian mosaic virus (MIMV; NC_036390), maize mosaic virus (MMV; NC_005975), physostegia chlorotic mottle virus (PhCMoV; KY859866), potato yellow dwarf virus (PYDV; NC_016136), rice yellow stunt virus (RYSV; NC_003746), sonchus yellow net virus (SYNV; NC_001615) and taro vein chlorosis virus (TaVCV; NC_006942). Cytorhabdovirus (red): alfalfa dwarf virus (ADV; NC_028237), barley yellow striate mosaic virus (BYSMV; NC_028244) and lettuce yellow mottle virus (LYMoV; NC_011532). Lyssavirus (black): rabies virus (RV; NC_001542). Perhabdovirus (violet): eel virus European X (EVEX; NC_022581). Figure S2. Comparisons between AaNV and selective members of the Nucleorhabdovirus genus. The consensus sequence of the intergenic conserved sequences (a), the 3′ and 5′ ends (b), and the genome organisation (c). The names and the accession numbers of the viruses can be found under Figure S1. Figure S3. Electron micrograph of the JKI-1607 reacting with AaNV ribonucleoprotein (RNP). a Enriched nucleocapsids after immunosorbent step; b Enriched nucleocapsids but not virions are covered (decorated) with antibodies (DOCX 1320 kb) [file 12985_2019_1147_MOESM1_ESM.docx]

**
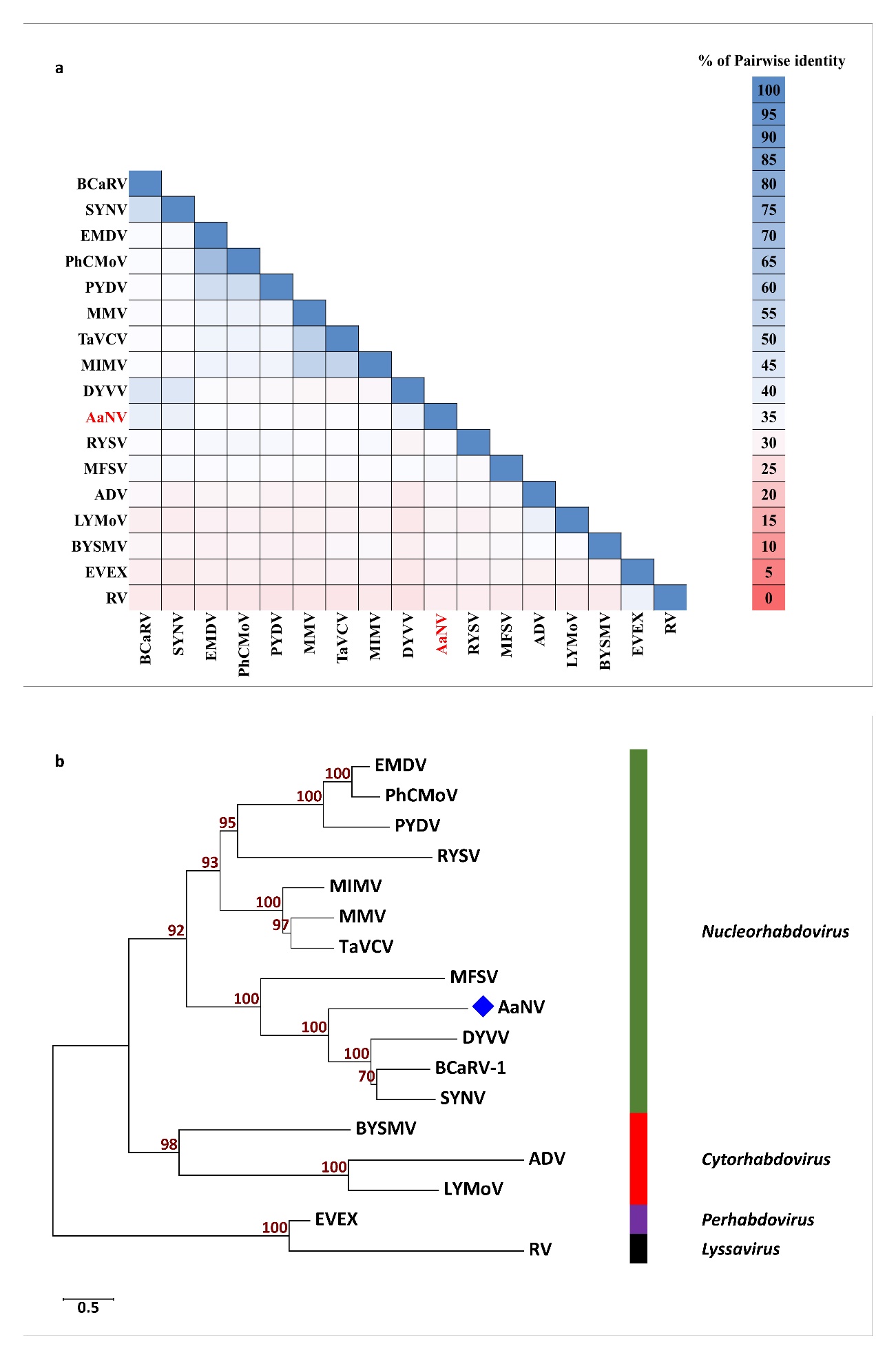
**

**Figure S1.** (**a**) Pairwise identity matrix of the whole genome sequences of AaNV with selected members of the family *Rhabdoviridae* (ClustalW 2.1); (**b**) Unrooted neighbour-joining phylogenetic tree [Genetic distance model (Jukes-Cantor) and 1,000 bootstrap replications] based on the nucleotide alignment of the whole genomes of AaNV and selected members of different genera of the family *Rhabdoviridae*. AaNV indicated by a blue solid diamond shape.

The names and the accession numbers of the viruses are as follow: *Nucleorhabdovirus* (green): alfalfa-associated nucleorhabdovirus (AaNV; MG948563), black currant-associated rhabdovirus 1 (BCaRV-1; MF543022), datura yellow vein virus (DYVV; NC_028231), eggplant mottled dwarf virus (EMDV; NC_025389), maize fine streak virus (MFSV; NC_005974), maize Iranian mosaic virus (MIMV; NC_036390), maize mosaic virus (MMV; NC_005975), physostegia chlorotic mottle virus (PhCMoV; KY859866), potato yellow dwarf virus (PYDV; NC_016136), rice yellow stunt virus (RYSV; NC_003746), sonchus yellow net virus (SYNV; NC_001615) and taro vein chlorosis virus (TaVCV; NC_006942). *Cytorhabdovirus* (red): alfalfa dwarf virus (ADV; NC_028237), barley yellow striate mosaic virus (BYSMV; NC_028244) and lettuce yellow mottle virus (LYMoV; NC_011532). *Lyssavirus* (black): rabies virus (RV; NC_001542). *Perhabdovirus* (violet): eel virus European X (EVEX; NC_022581).


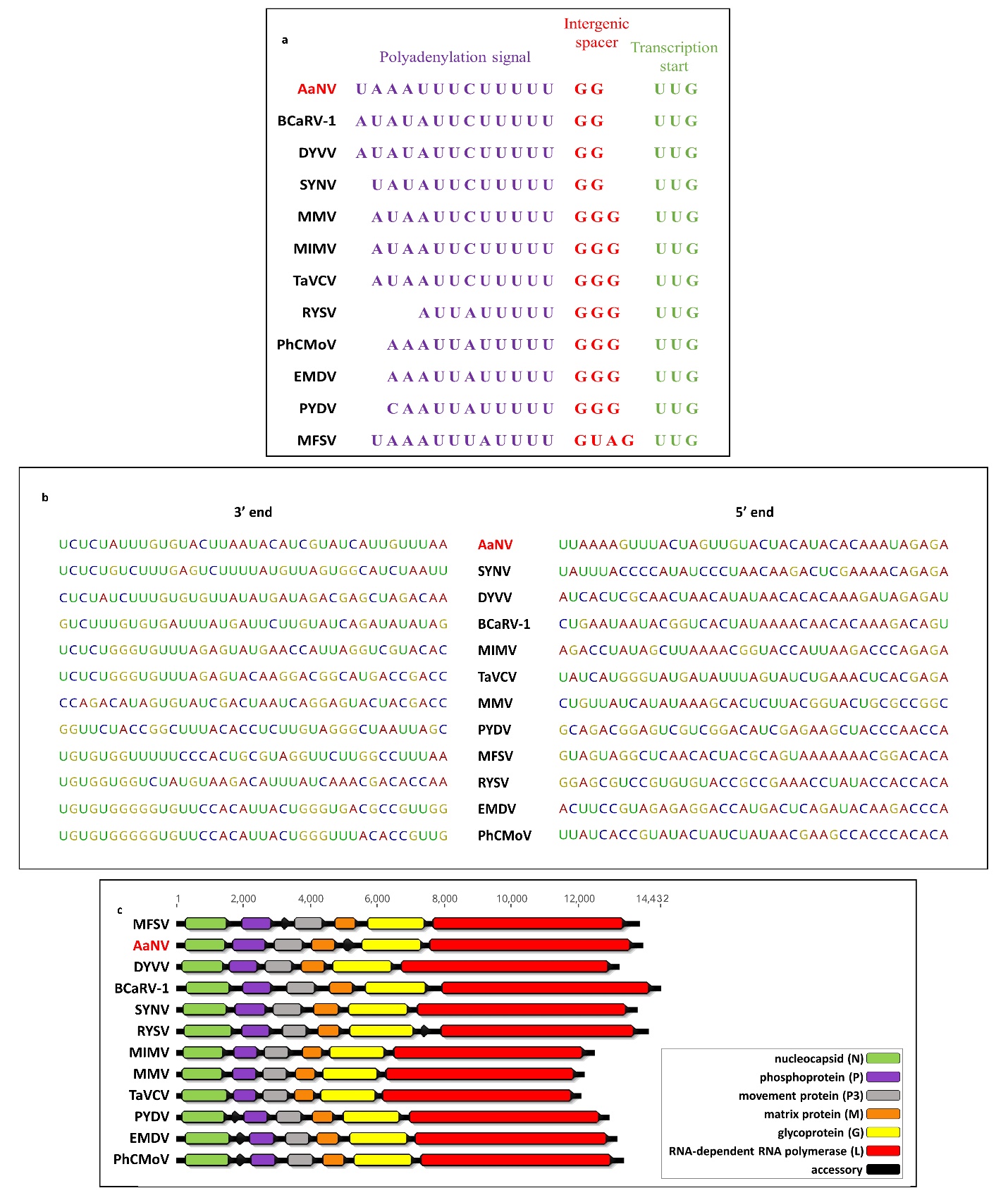


**Figure S2.** Comparisons between AaNV and selective members of the *Nucleorhabdovirus* genus. The consensus sequence of the intergenic conserved sequences **(a)**, the 3’ and 5’ ends **(b)**, and the genome organisation **(c)**. The names and the accession numbers of the viruses can be found under figure S1.

**
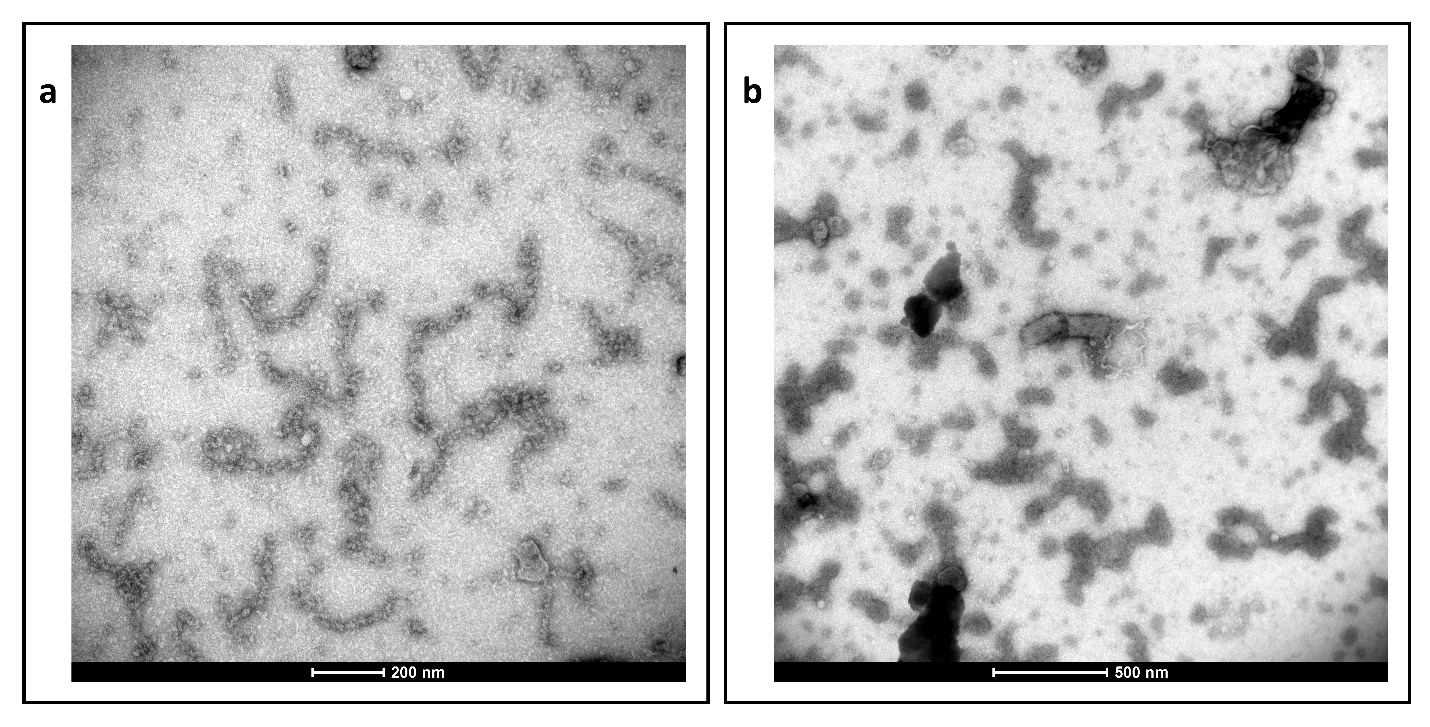
**

**Figure S3.** Electron micrograph of the JKI-1607 reacting with AaNV ribonucleoprotein (RNP). (**a**) Enriched nucleocapsids after immunosorbent step; (**b**) Enriched nucleocapsids but not virions are covered (decorated) with antibodies.
